# Emergence of Cooperative Impression With Self-Estimation, Thinking Time, and Concordance of Risk Sensitivity in Playing Hanabi

**DOI:** 10.3389/frobt.2021.658348

**Published:** 2021-10-12

**Authors:** Hirotaka Osawa, Atsushi Kawagoe, Eisuke Sato, Takuya Kato

**Affiliations:** ^1^ Human-Agent Interaction Laboratory, Faculty of Engineering, Information and Systems, University of Tsukuba, Tsukuba, Japan; ^2^ Human-Agent Interaction Laboratory, Graduate School of Science and Technology, University of Tsukuba, Tsukuba, Japan

**Keywords:** Hanabi, cooperative game, human-agent interaction, self-estimation, risk sensitivity

## Abstract

The authors evaluate the extent to which a user’s impression of an AI agent can be improved by giving the agent the ability of self-estimation, thinking time, and coordination of risk tendency. The authors modified the algorithm of an AI agent in the cooperative game Hanabi to have all of these traits, and investigated the change in the user’s impression by playing with the user. The authors used a self-estimation task to evaluate the effect that the ability to read the intention of a user had on an impression. The authors also show thinking time of an agent influences impression for an agent. The authors also investigated the relationship between the concordance of the risk-taking tendencies of players and agents, the player’s impression of agents, and the game experience. The results of the self-estimation task experiment showed that the more accurate the estimation of the agent’s self, the more likely it is that the partner will perceive humanity, affinity, intelligence, and communication skills in the agent. The authors also found that an agent that changes the length of thinking time according to the priority of action gives the impression that it is smarter than an agent with a normal thinking time when the player notices the difference in thinking time or an agent that randomly changes the thinking time. The result of the experiment regarding concordance of the risk-taking tendency shows that influence player’s impression toward agents. These results suggest that game agent designers can improve the player’s disposition toward an agent and the game experience by adjusting the agent’s self-estimation level, thinking time, and risk-taking tendency according to the player’s personality and inner state during the game.

## Introduction

An AI agent cooperating with a human player in a cooperative game cannot create a good gaming experience unless the human player sees the agent as a worthy partner. In this paper, we define a cooperative game as a game in which all players share a common score, and each player tries to raise the score. A human player who gets a good score in a cooperative game can not only understand the structure of the game well, but can also coordinate with the teammate that he/she understands the structure of the game. A good player in a cooperative game is considered to be a player who is thoughtful enough to read the intentions of the teammate and performs clear actions with intentions that can be easily interpreted by the teammate. An AI agent capable of such positive player actions is considered to contribute to cooperation in the sense of making a good impression on users, as well as directly contributing to scores. There have been many studies of algorithms in which AI agents and humans cooperate in human-agent interaction, but there are many unknowns about how the behavior of such algorithms improves the user’s impression.

In this study, we evaluate how giving an AI agent the ability to read the user’s intention improves the user’s impression, and how giving the AI agent behavior that is easy for the user to understand improves the user’s impression. We implement the algorithm of the AI agent in the cooperative game Hanabi to have both abilities, and investigate the change in the user’s impression by playing with the user. A self-estimation problem is used to evaluate the effect of the ability to read the intention of a user on the user’s impression. This problem estimates an uncertain agent state from the action of a user and compares the case of acting on the basis of this estimated state with the case of acting deterministically. The cognitive ability to infer a person’s thinking from their behavior is called social intelligence; complementing the self-state using social intelligence is considered to be a characteristic of human intelligence, and suggests cooperativeness (Luft and Ingham, 1961). In Hanabi, you can’t observe your own state by yourself, the player has no choice but to rely on information provided directly or indirectly by the other player’s actions. This study treats the latter role as a main ability of self-estimation.

In the evaluation of self-estimation, we set three conditions of two types for the agent and the human, depending on the presence or absence of another person’s observations and behavior simulation as teammates of the game, and analyzed the impression of the experiment’s participants. In our previous research, we assumed that games were played between agents using the same algorithm so that no error arose in the modeling of the other party (Osawa, 2015). In this study, as the agent’s teammate is a human being, potential error in modeling the teammate was also considered. Therefore, our analysis was limited to games in which simulations of observation and behavior of others would have a high probability of success.

We also implement the human type delay model to the agent by changing the length of the thinking time of the agent itself according to the action choice. We define the delay caused by the increase of the thinking time during complex thinking and processing as the human type delay. Thinking time can be a clue to a cooperative attitude. For example, the agent with more thinking time seems more cooperative when they express their opinions one after another without showing signs of being troubled during a discussion, or when they express their opinions one by one while being deeply troubled. People spend more time thinking and processing complex thoughts, and the more complex they think, the more likely they are to devote their resources to their collaborators. An attitude that does not aim to give the impression of being cooperative but gives such an impression as a result is defined as an implicit cooperative attitude in contrast to an explicit cooperative attitude such as “treat someone politely”. In the above example, it can be said that the tacit cooperative attitude is read from the information of the merits and demerits of the thinking time. We examine the effectiveness of a strategy to convey an implicit cooperative attitude of an agent to a human player by using a humanoid delay by actually playing a cooperative game with the human player.

In addition, a risk sensitivity matching task is used to evaluate the effect of the comprehensibility of the AI agent’s behavior from the user’s perspective on their impression. This task determines how an impression changes when the risk sensitivity of an agent’s action is changed according to the risk tendency of a user. We estimate that risk sensitivity matching is effective in improving user impressions based on the following reasoning: for example, a player can choose to inform the other player of a card and increase the other player’s probability of success in playing the card, or play his/her own card and accumulate the total score. Players who enjoy risk, even when their chances of success are low, focus on playing cards and accumulating points. Players who do not enjoy risk do not show their cards until they are well-informed and have a better chance of success. If only risk-inclined players play the game, or if only risk-averse players play the game, both score points and both parties are satisfied with the game result. However, consider a case in which player A, who likes risk, and player B, who does not like risk, play a game. Even if A tells B that the card should be able to be played with a given probability of success, B will not be inclined to play the card yet, so they will ultimately be dissatisfied. Thus, it is assumed that AI agents that can align with the risk sensitivity of human players will be considered by humans to be cooperative.

To verify whether an agent whose risk sensitivity is closer to that of the player improves the game experience for human players, two types of agents, one that prefers taking risks and another that avoids risks, were implemented for the cooperative game Hanabi. We examined whether a player’s impression of the AI agent was improved if its risk sensitivity was similar to that of the player in an experiment. First, the characteristics of the player were examined in terms of risk aversion and optimism/pessimism. Next, the player played Hanabi with a risk-inclined agent and a risk-averse agent. Finally, the relationship between the player’s personality and the impression value for each type of agent was examined.

The remainder of this paper is organized as follows. *Related Works for Agent Studies* explains related studies on how human impression improves cooperation and how motivative agents improve game play. *Hanabi* explains the background of the cooperative game Hanabi. *Effect of Self-Estimation* describes the evaluation of the effect of self-estimation. *Evaluation for Thinking Time* describes the evaluation of the thinking-time. *Effect of Risk Tendency* explains the evaluation of concordance of risk sensitivity. *Discussion* discusses the results of all evaluations. *Contribution* explains the contributions of our study, and our study’s limitations are explained in .*Limitation* Finally, *Conclusion* concludes the study.

## Related Works for Agent Studies

### Contribution of Agent’s Personality in Human Impression

The effects of personality similarities between people have been studied in the field of social psychology (Byrne, 1965). For example, Byrne et al. found that people who are interested in the same issues prefer each other. Such personalities have been found to be effective in AI agents as well as in humans (Liew and Tan, 2016).

To improve people’s impression of human-agent interaction, agents have also been able to mimic the characteristics of the human with whom they are interacting. For example, You et al. showed that the gender of the voice used for a robot and the degree of agreement between the opinions of the user and those expressed by the robot increase the user’s trust in the robot and willingness to cooperate with the robot (You and Robert Jr, 2018). Yonezu et al. found that when an avatar performs a synchronous action that mimics the nodding action and facial expression of a person during a remote conversation via a robot avatar, the human participant’s impression of the robot increases (Yonezu and Osawa, 2017). These studies have been conducted to improve impressions by synchronizing external characteristics and emotional expressions in visible agents.

### Agent Personality in Board Games

Board games demonstrate fundamental behaviors of human beings, and cooperative behavior during games is a task that requires intelligence and is intended for agent programs. Several studies in the field of artificial intelligence (AI) have attempted to solve various games. These challenges have been nearly resolved in complete information games. Programming acceptable game players has become a promising challenge in the field of game AI, and several games have started to focus on creating acceptable AI game players (Graepel et al., 2004; Soni and Hingston, 2008a; Chen et al., 2009; Nakamichi and Ito, 2015).

Researches have been conducted to enable game agents to provide a good game experience and motivation for players. Soni et al. insisted that the game agent should not only seek the optimum solution to defeat the human, but should act in a way that increases the human player’s enjoyment, and implemented a game agent in a first-person shooter game (Soni and Hingston, 2008b). It was found that the customized game agent made the player want to continue playing the game. Sephton et al. implemented a game agent more suitable for entertainment by changing the strength of the agent in Lords of War, a strategic card game (Sephton et al., 2015). Fujii et al. state that enemy agents in Super Mario can entertain human players by improving their ability to defeat humans while learning near-human behavior (Fujii et al., 2013). The above studies determined how to motivate and entertain the player through the implementation of a competitive game agent that adjusted to human ability.

## Hanabi

### Background of Hanabi Study: A Unique Testbed for Analyzing Human Cooperation

Research on AI agents playing Hanabi has been widely conducted in recent years (Osawa, 2015; Cox et al., 2015; Walton-Rivers et al., 2019; Sato and Osawa, 2019; Bard et al., 2020). Hanabi is a game where the results tend to differ depending on the combination of the teammate’s strategy and your own. From 2018, a competition was held as a part of the Computational Intelligence and Games (CIG), an international conference on computer games (Walton-Rivers et al., 2019). There are two types of competitions: one in which the same agents collaborate with each other, and the other in which different agents collaborate with each other. A game played between similar agents is suitable for obtaining a theoretical solution. One of the most famous studies examining Hanabi’s theoretical solutions was the work of Cox et al. (2015). They took Hanabi’s problem as a hat guessing task (Butler et al., 2009) and found that they got an average score of 24.7 in a five player game. Hanabi is a game with a maximum score of 25, so that’s close to perfect. Bouzy also found that teaching tips in a more diverse way increased the score to 24.9 (Bouzy, 2017). On the other hand, a game in which different agents cooperate with each other is excellent for dealing with issues related to agent “theory of mind” and “cooperation” such as the intention recognition (Walton-Rivers et al., 2017; Rabinowitz et al., 2018).

One of unique feature of Hanabi game is that this game requires cooperation without an alpha player (Engelstein and Shalev, 2019). The alpha player problem or the magistrate problem is a problem in which a cooperative game becomes substantially a one-player game. In general, cooperative games allow a group to act more efficiently by putting one person as a leader and aggregating information there. However, if this is done, only the alpha player makes a decision, and the other players become only the slave players, and those players lose interest in the game. Hanabi excels at solving these problems with rules. Each player has no information about their own cards. He or she must be told about it by others. In addition, since there are restrictions on how to teach the information on the card and it is necessary to pay a cost to teach, it is consequently inefficient to create a magistrate.

Hanabi is also unique in that there is no communication element and only guesses the intentions of the other person. This point was also mentioned in the study by Bard et al. which highlighted the Hanabi study (Bard et al., 2020). In general, when dealing with communication in multi-agent research, we assume a mechanism called “cheap talk” before negotiation between agents (Lewis et al., 2017). There is no cost in exchanging information during the Cheap talk phase. But with Hanabi, you have to read the intent from the other player’s card without the cheap talk. Hanabi is an interesting challenge in that it requires reading intention just by the exchange of costy information. It’s also a computationally manageable game challenge in that it does not have to deal with natural language.

Hanabi is used as a good testbed for agents which improves the result by changing its own parameter and algorithm by the behavior of the partner. Sato et al.'s study showed that Hanabi agents can reduce cognitive load by mimicking the play timing of human players (Sato and Osawa, 2019). Liang et al. showed that agents who use conversational implications and choose behaviors that emphasize the information given to the turn, and agents who emphasize narrowing the card’s potential, are perceived as “human” who tend to feel human (Liang et al., 2019). Canaan et al. suggested using a simulation of the play between AI, that the diversity of the player could be correspondent, if 2 parameters of “risk aversion” and “degree of communication” were set appropriately (Canaan et al., 2019). This study verifies whether AI agents with different tendencies of “risk aversion” can be implemented and actually adapted to human diversity. Eger explained that people who play the card game Hanabi highly evaluate AI agents who feel like they are acting with intention (Eger et al., 2017). Gottwald et al. devised the agent which got information on what kind of intention is going to transmit from eye movement of the human player in Hanabi (Gottwald et al., 2018).

### The Rules of Hanabi

Hanabi is a 2–5 person turn-based card game. The total number of cards is 50, each with a color and a number. The colors are white, red, blue, yellow, or green and the number is 1, 2, 3, 4, or 5. Each color has 3 sets of 1, 2 sets of 2–4, and 1 set of 5. In addition, eight information tokens are used. The purpose of this game is to arrange as many cards as possible in the order of 1, 2 up to 1–5 cards of each color on the board, and the total number of cards arranged at the end of the game is the score.

At the start of the game, each player draws 4 (when four or five people play) or 5 (When two or three people play) cards from the shuffled deck of 50 cards. The player plays the game by pulling the hand from the deck to maintain the initial number of cards. An important feature of this game is that each player cannot see the contents of his or her own hands, but they can see the contents of other players’ hands.

**FIGURE 1:**
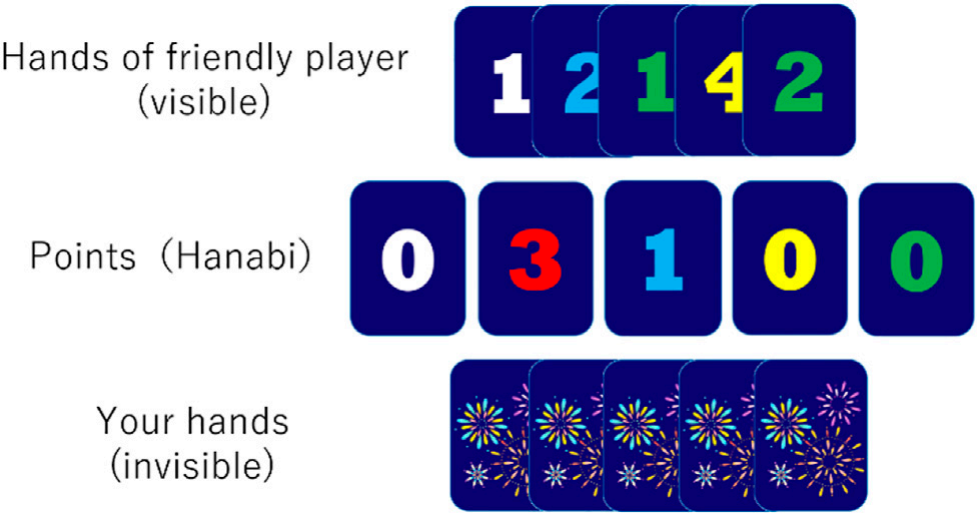
An example situation in Hanabi (extracted from developed Interface).

Each player must do one of the following actions in one turn:

• Give information
  ➢ The player can give information of the other player’s hand to him or herself. The player can give either color or number information. For example, information such as “The yellow cards are the second and third from the left.” and “You have a card with 2 that is the first one from the left.” can be given.
  This action consumes one information token.
• Discard a card
  ➢ You can throw away a card from your hand. All discarded cards will be shown to all players. The discarded cards do not return to the deck. This action increases the number of information tokens by one if they are less than eight.
• Play a card
  ➢ You can play a card from your hand. The “play” mentioned here is an act of taking out a card from one’s hand and checking whether the card is a playable card. This can be done whether or not the information on the card being played is known. This action cannot be undone. If the card played is a playable card, a score is added and the Hanabi board is updated. For example, in the situation shown in Figure 1, if the hand played by the player is one of white 1, red 4, blue 2, yellow 1, and green 1, the play is successful. Otherwise, play fails.

There are three exit situations for the game.

• Get 25 points
  ➢ 25 points is the highest score in the game, so the game ends.
• Deck becomes empty
  ➢ Each player has one turn of their own after the deck has been emptied.
• Three failures to play cards in the game
  ➢ The game ends.

In our study, the score becomes the result at the end of the game, when it finishes under this condition. In addition, in the official rule, the score becomes 0 when it finishes under this condition, but it was changed to the above rule for the sake of simplicity.

### Implementation of Base Algorithm

**FIGURE 2:**
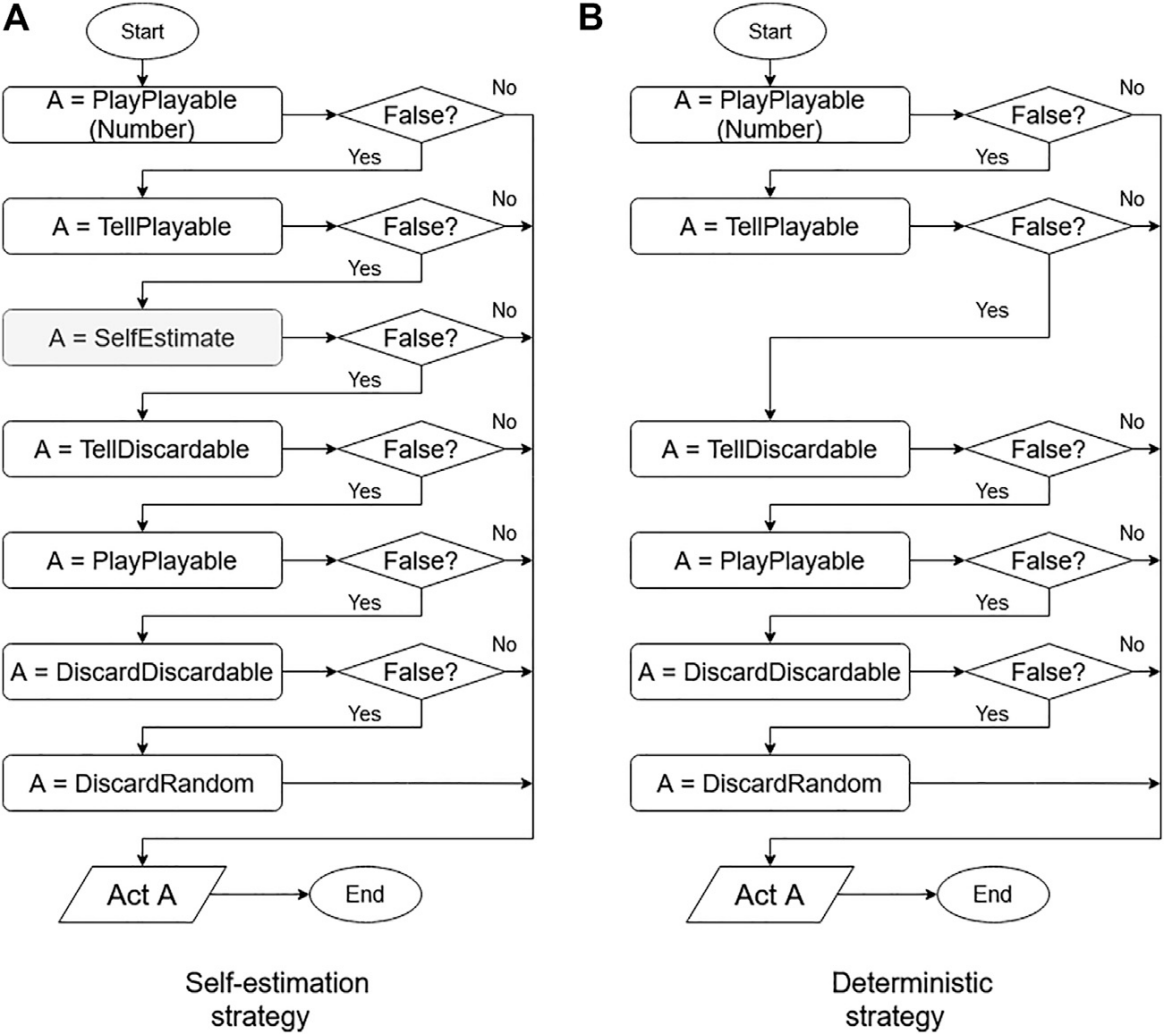
Flowcharts for self-estimation and deterministic strategies.

The conventional deterministic strategy is the algorithm proposed by Osawa (Osawa, 2015), which acts with the following priorities. This method is also widely used as a reference in other Hanabi studies (Canaan et al., 2019). Each process is conducted according to Figure 2B.

#### PlayPlayable: Play Playable Card

In this action, the agent determines the success probability of playing the card in their hand from the card information and the current state of the Fireworks field. If the agent knows that a playable card exists, based on the information of its hand and the observable cards on the board, the player plays the card.

For example, if the contents of the card are known to the agent, it is clear whether the card is playable by looking at the score of the field. Assuming that the content of one of the agent’s cards is determined to be a red 3 from the information given by the other player, if the red Firework on the field is 2, then the play success probability for that card is 100%. Also, even if the information of the card is not determined, it may be determined that the play of the card is successful. Suppose you have a card in your agent’s hand that you know the number is 1, but you don’t know whether the color is blue, red, or yellow. At this time, if the blue, red, and yellow Fireworks of the field are 0, the agent determines that the card known to be written as 1 is surely a card to be successfully played.

In the *Effect of Self-Estimation* experiment, the goal was to play more like humans. To do this, we watched people play, divided PlayPlayable into two stages (PlayPlayable with numbers and PlayPlayable with other information), looking at the numbers and letting them play if they could. If the agent can’t play a card based only on number information (disregarding colors), the agent gives priority to giving information. However, we found that the process itself had little impact on the results, so we made it a simpler condition in *Evaluation for Thinking Time* and later experiments.

#### DiscardDiscardable: Discard Unnecessary Card From Agent’s Own Hand

This function selectively discards unnecessary cards after the turn. “Card is Unnecessary” means that the card has already appeared on the field as Hanabi, so it is not subject to an increase in score. For example, suppose you have a card that the agent knows is a blue 2, and the blue Hanabi in the field is 4. At this time, the blue 2 card is unnecessary, and it is possible to discard the card. It is also important that IF all connection cards are discarded, preceding cards are also dicardable. For example, all green 4 are discarded, a green 5 card is also discardable.

#### TellPlayable and TellDiscardable: Inform Other About Information of Cards

This action provides information when it is found that a teammate has a playable card or unnecessary card, and information regarding the card is incomplete for the teammate. This function can only be performed when the information token is on the field. The agent provides the number or color information of a playable card to a friendly player. Basically, number is more important than color information and it is sometimes informed first. Whether the player’s card is playable is determined in the same way as in “PlayPlayable.”

#### Other Random Actions

Other actions used in Hanabi games are as follows.

• TellRandom: This function can only be performed when the information token is in the field. In this action, the agent gives random numbers or color information to the teammate.
• DiscardRandom: This function discards cards randomly selected from the agent’s hand.

## Effect of Self-Estimation

In this section, we describe an algorithm that simulates others’ observations for self-estimation, an agent that performs self-estimation, and an agent that does not perform self-estimation.

### SelfEstimate Action: Estimation Process for Own Hands From Teammate’s Action

An agent using the self-estimation strategy converts its hand into a combination of possible cards and compares the teammate’s simulated action and actual action at this time to determine a possible set of estimated agent’s hand. We constructed our algorithm based on our previous work (Osawa, 2015). We used outer-state strategy on above paper as the base of deterministic strategy in this paper, and used self-recognition strategy on above paper as the base of self-estimation strategy. As explained in *PlayPlayable: Play Playable Card* the information phase from the agent is separated in two sections as number and colors. Other conditions are same as our previous paper.

First, the agent considers a possible combination of cards as a possible candidate for its own hand. Possible cards are those which match the known information. The agent obtains the simulated result of the teammate’s action using the simulation of the teammate’s observation, which occurred in the latest turn and is generated from the hypotheses regarding possible cards, as well as the current board. To simulate this action, we used our own action decision algorithm.

The agent estimates its hand for each of the five cards. For cards with incomplete information regarding the color or number, the elements of the card in the estimated hypotheses (possible combinations) are sorted by the number of occurrences which means how many times each combination is expected. When the value of the largest number of occurrences is greater than or equal to 1.8 times the value of the next largest occurrence count, that card is estimated as the most frequent element. The value of 1.8 is empirically determined. When the elements of the estimated card do not overlap with the player’s hand, this estimation is applied. If the value is not larger than 1.8. the selfEstimate simple fails.

We want to explain it in detail on here. If the agent only has cards with two greens on left and other information is unknown, the table has yellow and green 1 s Fireworks, and the partner discard his/her own card in previous turn. The agent cannot directly see its cards and the agent can simulate that every combination of its card patterns that might be saw by the partner. If the agent simulates the world that the agent has green 2 on left, the agent can estimate that the world is impossible because the agent’s partner does not notify that call the agent’s a number of the card. It is also impossible to estimate that the agent has green 1 because the partner will inform the agent its discardable card by number in that case. On the other hand, the agent can count the world that the agent has green 3 on the agent’s deck because in that case, the partner is possible to discard the agent’s own card. So, the agent can count all possible world that left card is green 3 in case.

Like above process, the agent with SelfEstimate can collect set of all possible worlds and count how many cases are matched in each card based on previous actions, not only discard, but all actions. And as a result, if top case is 1.8 larger than second case, the agent estimate that the card information is believable.

### Classification of the Agent’s Behavior

The elements in the flowchart shown in Fig. Figure 2 follows. The box labels show the functions explained in *Implementation of Base Algorithm.* The return value of a function can be either true or false. Each function stores the agent’s behavior. Returning true means that the action stored in the function can be performed on that turn by the agent; otherwise, the function returns false. The agent behavior that is stored in each function is explained in the following.

The left side of Figure 2 shows the algorithm with self-estimation, while the right side shows the deterministic algorithm. In the gray box labeled SelfEstimate, the self-estimation algorithm described in *SelfEstimate Action: Estimation Process for Own Hands From Teammate’s Action* is applied. An agent using the self-estimation strategy performs actions based only on deterministic information, and uses probabilistic strategies only for self-estimation. To provide a comparison of the estimation strategies, an agent not using the estimation function used in *SelfEstimate Action: Estimation Process for Own Hands From Teammate’s Action* is presented here. This agent performs actions based only on deterministic information.

### Evaluation of Self-Estimation

In a game between humans and agents, experiments were conducted to evaluate human players’ perception of human-like self-estimation behavior in the agent in terms of human thought, impression, and game impact.

To investigate the influence of the subject’s actions and impressions on his/her partner, video recording and an impression evaluation questionnaire were used to record the state of the subject in addition to his/her game record. The questionnaire contained the following items:

• Impression of the teammate
  ➢ Q1. Did you feel that the teammate has its own will?
  ➢ Q2. Did you feel that the teammate is familiar with games?
  ➢ Q3. Was the teammate friendly?
  ➢ Q4. Did you feel that the teammate considered your actions?
  ➢ Q5. Was the teammate wise?
  ➢ Q6. Was the oppoent acting according to your intention?
  ➢ Q7. Were you aware of the intention of your teammate’s actions?
• Impression of the game
  ➢ Q8. Were you trying to raise the score?
  ➢ Q9. Was the game easy to play?
  ➢ Q10. Do you feel that you made mistakes?
  ➢ Q11. Did you play the game in a initiative role?
  ➢ Q12. Are you satisfied with the results of the game?
  ➢ Q13. Please provide any additional comments (a free description field was provided)

Q1 to Q5 refer to Bartneck’s *Godspeed Questionnaire Series* (Bartneck et al., 2008). For game partners, Q1 and Q2 examined anthropomorphic qualities, Q3 and Q4 concerned likability, and Q5 was intended to measure perceived intelligence. Q6 and Q7 were intended to check whether communication with the teammate was planned. Q8 to Q12 were intended to gather information about players’ impressions of the game when playing with different teammates. All the questions were evaluated using a 7-point Likert scale [from “I disagree” (1) to “I agree” (7)]. This questionnaire was given to the users at the end of each game, and an interview was conducted at the end of each experiment. The differences between the time taken by the models and the time taken by the participant to complete an action were recorded as “thinking time.”

The experiments were conducted using two pairs of participants. The two subjects were briefed about Hanabi’s rules and interface manipulation methods. The experiments were then conducted in different rooms. The participants played the game six times. Three games of each strategy were played: one set with agents based on the self-estimation strategy (hereinafter referred to as the “S condition”), one with agents based on a deterministic strategy (hereinafter referred to as the “D condition”), and one with a human-human condition (hereinafter referred to as the “H condition”). Then, the game was played again once for each strategy in the same order as in the first three conditions. Six different types of card sets were prepared so that one subject could play six games with six types of decks. In addition, considering the learning effects of the subjects’ games, counterbalancing was ensured by equally allocating the order of conditions among all subjects. We conducted a questionnaire after each game and ended the experiment as soon as we completed the interviews with the questionnaire for the sixth game.

Twelve participants who had never played Hanabi participated in the experiment. A total of 12 undergraduate and graduate students in their 20s (12 men) participated in the experiment.

### Hypothesis

In this experiment, it was assumed that the agent’s self-estimation caused the subject to think that the agent was a human. Furthermore, when the self-estimation succeeded, the subject participated together with the agent and became familiar with the agent and the game. In our previous research on agent simulation, we showed that an agent’s self-estimation function significantly affects the score of the game (Osawa, 2015).

Based on the above, we assumed the following as a hypothesis.

1 The agent using the self-estimation strategy will score higher on Q1, while the agents using the deterministic strategy will score higher on Q2.
2 If the success rate of self-estimation is high, the impression evaluations for Q3 to Q7 will be higher than those for the deterministic agents and the score will rise.

### Result

We performed 24 experiments and obtained 23 sets of data, excluding one error. No cards were played in one of the S-condition games, and as no information was available for blind self-estimation, it was excluded from the analysis.

The self-estimation is conducted averaged 5.6 times (SD: 3.4) per game, and the percentage of cards that were correctly estimated was 63% on average (SD: 24) per game. In addition, estimated cards were played an average of 3.7 times (SD: 2.4) per game, while the percentage of success in playing cards was 63% on average (SD: 38) in one game.

**FIGURE 3:**
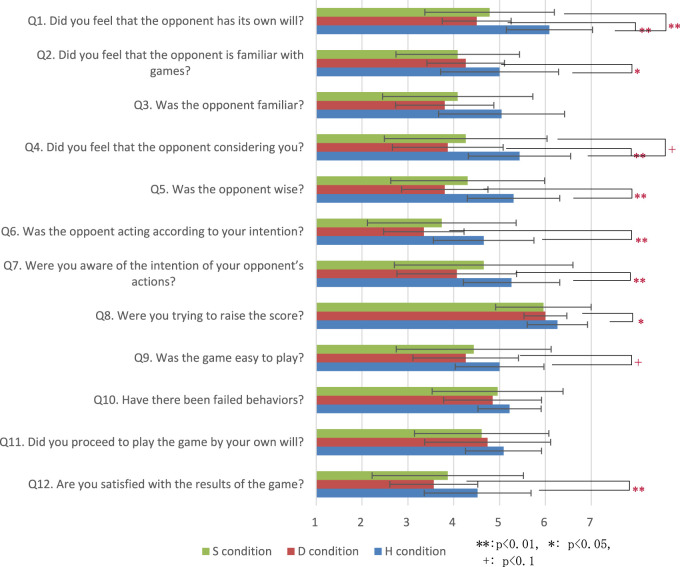
Questionnaire results evaluating impression of S, D, and H conditions.

Because self-estimation strategies can fail, it is necessary to extract successful S conditions. We classify successful S conditions as S′ conditions, wherein the success rate of self-estimation by the agent among the games of the S condition is high. To investigate the influence of the D and H conditions, multiple analyses were conducted with regard to impression evaluation, score, and thinking time. S′ condition games were classified as games in which the modeling and estimation of the teammate were correctly performed and the success rate for playing cards (estimated by self-estimation) was greater than or equal to 0.5, according to the requirements of an S condition game. The results of the experiment showed that 17 of the 23 S condition games could be classified as belonging to the S′ condition. We also 17 of 23 D/H condition games as D′/H′ condition games that joined participant on S′ conditions Figure 3.

#### Evaluation for Impression

**FIGURE 4:**
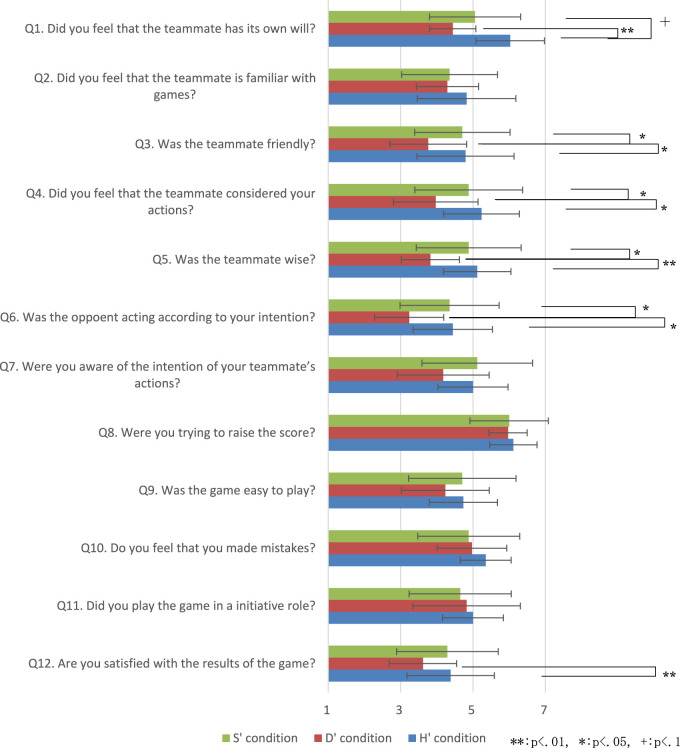
Questionnaire results evaluating impression of S′, D′, and H′.

We analyzed the results of multiple comparison tests using the Bonferroni method on the differences in each evaluation item for the three conditions shown in Figure 4 and Table 1. The test results confirmed significant differences between the S and H conditions for Q1. The test results also confirmed significant differences between the D′ and H′ conditions for Q1, Q2, Q4, Q5, Q6, Q7, Q8, and Q12. The test results confirmed significant differences between the S′ and D′ conditions for Q3, Q4, Q5, and Q6. The test results also confirmed significant differences between the D′ and H′ conditions for Q1, Q3, Q4, Q5, Q6, and Q12. There is less significant difference between S condition and D condition than S′ condition and D′ condition because S condition which self-estimation is not applied cases, it is not different from D condition.

**TABLE 1:** Result of questionnaire in constant thinking time experiment.

	C condition average (conventional)	P condition average (present)	*p* (*: *p* < 0.05)
Q1	4.05 (SEM 0.308)	4.98 (SEM 0.263)	1.71e-3^*^
Q2	4.43 (SEM 0.267)	4.65 (SEM 0.251)	0.302
Q3	4.03 (SEM 0.275)	4.28 (SEM 0.252)	0.523
Q4	4.75 (SEM 0.284)	4.65 (SEM 0.241)	0.836
Q5	4.80 (SEM 0.272)	5.00 (SEM 0.242)	0.579
Q6	3.75 (SEM 0.284)	3.95 (SEM 0.255)	0.272
Q7	3.53 (SEM 0.255)	3.70 (SEM 0.243)	0.465

#### Score and Thinking Time

The scores are 19.76 (SD 0.64) in S′, 17.70 (SD 1.8) in D, and 17.61 (SD 4.0) in H. Multiple comparisons of the differences in the scores of the three conditions using the Bonferroni method showed no significant difference between the S and D conditions. The results of the tests comparing the S and D conditions indicated no significant differences between the cases when the score was derived by an agent using the self-estimation strategy with a high success rate.

Thinking time was considered as the average thinking time used for one action during the game. Using the Bonferroni method, we conducted multiple comparisons of the differences in the average thinking time in the three above-mentioned conditions. The results showed no significant differences between the S and D conditions. Moreover, no significant difference between the S and D conditions was noted for average thinking time when the consideration of agents using the self-estimation strategy was limited to those with a high success rate.

## Evaluation for Thinking Time

### Implementation of Thinking-Time

In addition to the self-estimation algorithm shown in Figure 2 left, we designed an algorithm to intentionally lengthen the thinking time when performing a low-priority action. By adding this function, a human-like agent which changes the merits and demerits of the thinking time by the action selection can be implemented. Specifically, the time from the time when the human player determines the action to the time when the agent determines the action is usually set to 3.5 s, but 7.5 s in the case of providing random information, and 9.5 s in the case of random disposal. These actions have low priority even in an actual human player and are hesitant to execute, and it is considered that by prolonging the thinking time in such actions, a cooperative attitude can be shown to the other party.

In this study, we conducted experiments comparing agents with constant thinking time regardless of behavior and agents with random thinking time regardless of behavior. Each of them is a constant thinking time experiment and a random thinking time experiment.

### Experimental Procedure for Comparison With Constant Thinking Time

A total of 20 undergraduate and graduate students in their 20s (11 men and nine women) participated in the experiment. After learning about Hanabi’s rules and interface, participants practiced once and then played the game four times. A game with a conventional deterministic agent in which the thinking time is constant regardless of the action selection (hereinafter referred to as C condition) and a game with a new deterministic agent in which the thinking time is changed according to the action selection (following P conditions) were played 2 times each for a total of 4 times. Each time the game was over, a questionnaire was conducted, and finally an interview was conducted to complete the experiment. Interviews after the four games were conducted mainly to find problems with the experimental procedures. Unlike questionnaire, there were no clear questions in advance. There were no major problems found in the interview.

Even in the constant thought time experiment, the game record and the impression evaluation questionnaire after the experiment were carried out. Below are the questions from the questionnaire.

• One’s impression of the opponent
  ➢ Q1. Did you feel that the opponent was worried a lot?
  ➢ Q2. Did you think that the opponent is clever?
  ➢ Q3. Did you feel the opponent friendly?
  ➢ Q4. Did you understand the intent of the opponent’s actions?
• One’s impression of the game
  ➢ Q5. Was the game easy to play?
  ➢ Q6. Do you feel familiar with the game?
  ➢ Q7. Are you satisfied with the results of the game?

All questions were answered in the same manner as in the preliminary experiment, using a seven-point Likert scale.

**FIGURE 5:**
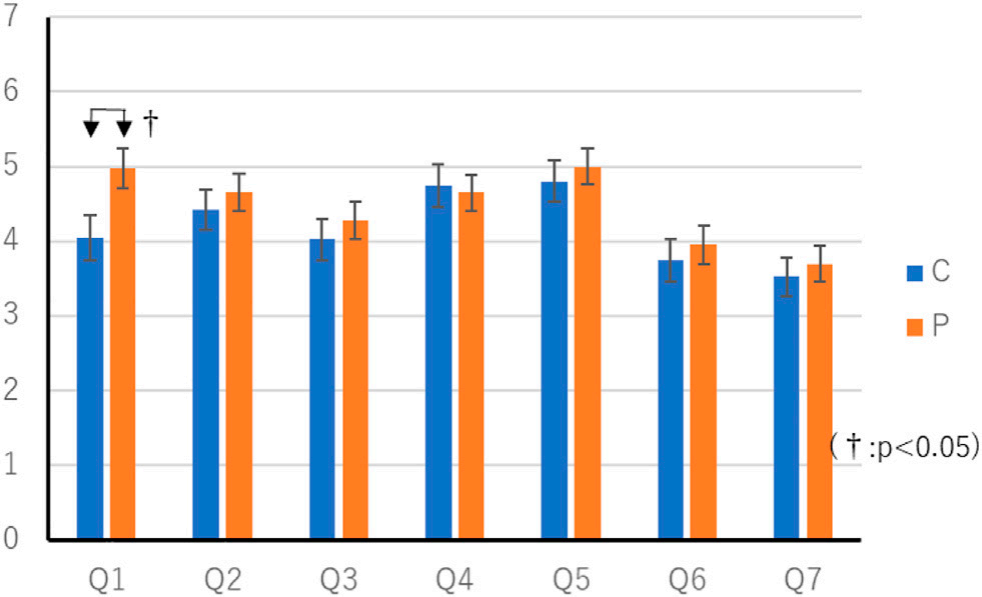
Result of questionnaire in Constant thinking time experiment.

As for the items of the questionnaire, Q1–Q3 were questions concerning the feelings of agents. Depending on how far away you are from the middle, the agent’s emotional diversity, or humanity, is measured. After the game with each agent was over, the items of the flowchart about the algorithm of the agent were filled out in the form of selecting an arbitrary number by ordering from the eight actions specified by us. This is to assess how well a player understands an agent’s strategy and how smart the agent is by how complex the strategy is. The more complex the strategy and the more items on the flowchart, the smarter the player is likely to evaluate the agent. We also asked which agent was easier to play with after all the games had ended Figure 5.

### Result of Comparison With Constant Thinking Time

The average score in the C condition was 16.1 (SEM 0.48) points, and the average score in the P condition was 16.3 (SEM 0.24) points. Wilcoxon’s rank-sum test showed no significant differences in scores. The questionnaire results of the constant thinking time experiment are shown in Tables 2, 3. Table 1 Wilcoxon’s rank-sum test showed a significant difference in “Q1. Did you feel that you were worried a lot?” (*p* < 0.05).

**TABLE 2:** Comparison test results between the S′, D, and H conditions.

	Difference in average value **: *p* < 0.01, *: *p* < 0.05		
	S′ condition–D condition	S′ condition–H condition	D condition–H condition
Q1	0.618	−0.971	−1.588*
Q2	0.059	−0.471	−0.529
Q3	0.941*	−0.088	−1.029*
Q4	0.912*	−0.353	−1.265*
Q5	1.059*	−0.235	−1.294**
Q6	1.118**	−0.088	−1.206*
Q7	0.941	0.118	−0.824
Q8	0.029	−0.118	−0.147
Q9	0.471	−0.029	−0.500
Q10	−0.088	−0.471	−0.382
Q11	−0.176	−0.353	−0.176
Q12	0.676	−0.088	−0.765*

**TABLE 3:** Means of evaluations of each agent and *p*-value of their *t*-test (N: 30)^*^: *p* < 0.05.

Q on agents	Mean (H)	Mean (L)	*p*-value
QA-1	4.63	4.90	0.49
QA-2	4.10	4.27	0.68
QA-3	4.77	3.93	0.03^*^
QA-4	3.23	3.57	0.45
QA-5	4.10	3.60	0.21
QA-6	4.63	4.20	0.35
QA-7	4.00	4.13	0.77

**FIGURE 6:**
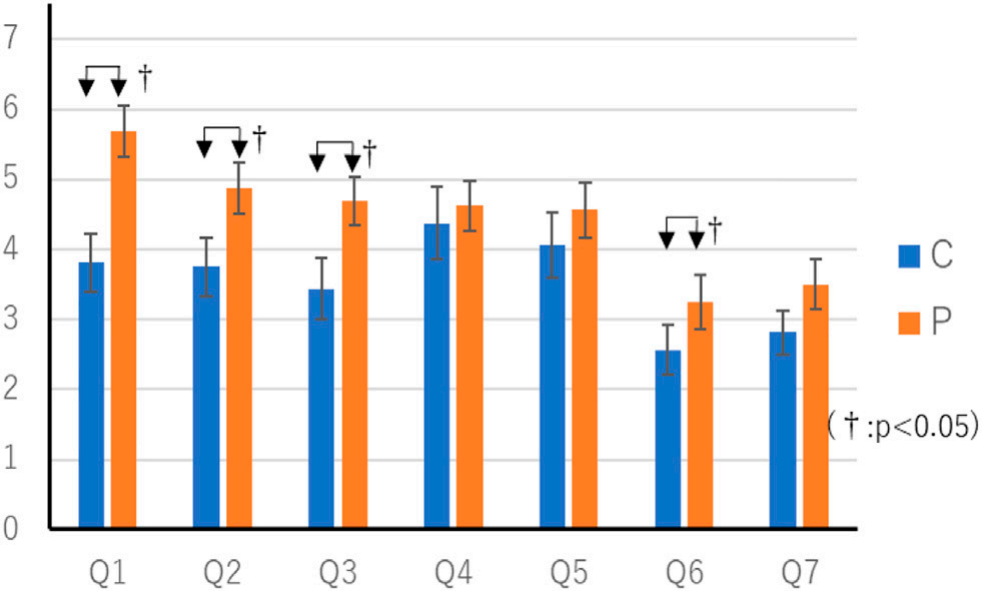
Result of questionnaire whose players notice difference in length of thinking time.

Also, 8 out of 20 people noticed that the two agents’ thinking time was different during the questionnaire and interview stages. The results of this survey are shown in Table 4 and Figure 6. Wilcoxon’s rank-sum test showed a significant difference among the eight patients in “Q1. Did you feel that you were worried a lot?” “Q2. Thought you were clever.” “Q3. Do you feel friendly?” “Q6. Do you feel familiar with the game?” (*p* < 0.05). There was no difference in the number of items in the flowcharts prepared by the participants between the two conditions. Six persons answered that the agent with the condition C was better, 12 persons answered that the agent with the condition P was better, and two persons answered that there was no difference in either. Fisher’s exact test showed no significant differences.

**TABLE 4:** Result of questionnaire whose players notice difference in length of thinking time.

	C condition average (conventional)	P condition average (present)	*p* (*: *p* < 0.05)
Q1	3.81(SEM 0.417)	5.69(SEM 0.363)	3.05e-4^*^
Q2	3.75(SEM 0.419)	4.88(SEM 0.350)	0.0225^*^
Q3	3.44(SEM 0.442)	4.69(SEM 0.352)	0.0166^*^
Q4	4.38(SEM 0.514)	4.63(SEM 0.395)	0.627
Q5	4.06(SEM 0.463)	4.56(SEM 0.380)	0.464
Q6	2.56(SEM 0.353)	3.25(SEM 0.354)	0.0977^*^
Q7	2.81(SEM 0.321)	3.50(SEM 0.548)	0.192

### Experimental Procedure for Comparison With Random Thinking Time

In the constant thinking time experiment alone, it is not clear whether the factor that the agent changes the thinking time according to his/her own action conveys the cooperative attitude, or whether the factor that the agent’s thinking time changes only randomly changes the player’s impression. Therefore, we conducted a random thinking time experiment in order to examine in more detail how the change in thinking time according to the agent’s action affects the player.

Of the 20 participants in the constant thinking time experiment, 15 (Nine men, six women.) participated in the random thinking time experiment. The participants practiced once and then played the game four times. In this experiment, we played a game with a new deterministic agent (following P conditions), which changes the thinking time according to the behavior selection as in the P condition of the constant thinking time experiment, and a game with an agent (following R conditions), which calculates the ratio of the thinking time of the agent based on the result of the constant thinking time experiment and randomly decides the thinking time regardless of the behavior selection based on the ratio, two conditions were respectively played 2 times.

For this second experiment, I told them in advance that the algorithms of the two agents are the same, and only the way of changing the thinking time is different so that the result does not depend on whether or not they remember the contents of the previous experiment. Each time the game was over, a questionnaire was conducted, and finally an interview was conducted to complete the experiment.

### Result of Comparison With Random Thinking Time

**FIGURE 7:**
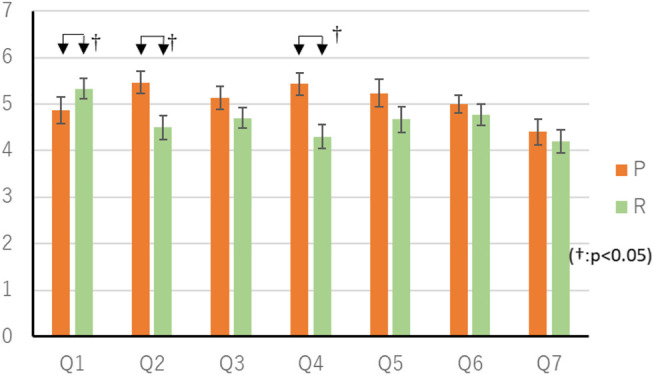
Result of questionnaire in Random thinking time experiment.

The mean score in the P condition was 16.1 (SEM 0.65) points, and the mean score in the R condition was 16.0 (SEM 0.49) points. Wilcoxon’s rank-sum test showed no significant differences in scores. The questionnaire results of the random thinking time experiment are shown in Table 5 and Figure 7. Wilcoxon’s rank-sum test showed a significant difference in “Q1. Did you feel that you were worried a lot?” “Q2. Thought you were clever.” “Q4. Do you understand the intent of your actions?” (*p* < 0.05). There were 11 respondents who preferred the P-condition agent, three respondents who preferred the R-condition agent, and one person who agreed that there was no difference. Fisher’s exact test showed no significant differences.

**TABLE 5:** Result of questionnaire in random thinking time experiment.

	C condition average (conventional)	P condition average (present)	*p* (*: *p* < 0.05)
Q1	4.87(SEM 0.293)	5.33(SEM 0.218)	4.40e-2^*^
Q2	5.47(SEM 0.234)	4.50(SEM 0.257)	7.48e-4^*^
Q3	5.13(SEM 0.257)	4.70(SEM 0.217)	0.190
Q4	5.43(SEM 0.239)	4.30(SEM 0.259)	2.80e-4^*^
Q5	5.23(SEM 0.297)	4.67(SEM 0.280)	0.138
Q6	5.00(SEM 0.183)	4.77(SEM 0.229)	0.283
Q7	4.40(SEM 0.277)	4.20(SEM 0.256)	0.419

## Effect of Risk Sensitivity

### Implementation of Risk Sensitivity

**FIGURE 8:**
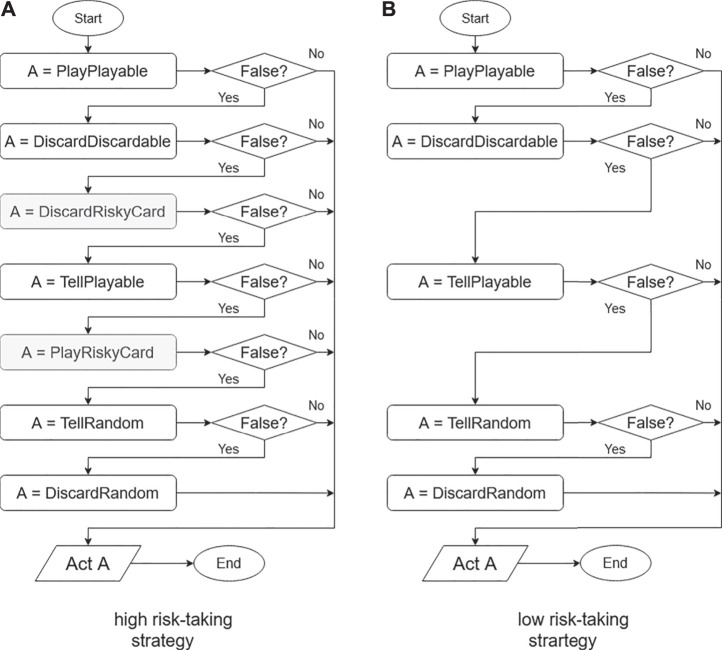
Flowcharts for risk-tendency strategies.

We implemented two rule-based agents with different risk-taking tendencies by modifying our previous implementations. The high risk-taking strategy is shown on the left in Figure 8, and the low risk-taking strategy is shown on the right.

In this experiment, the order of the parts of the flow chart is different from that in Figure 2, a change mainly intended to examine the effects of risk trends. We also added two more actions, shown as gray boxes.

#### DiscardRiskyCard

This is an action to discard cards that do not contribute more than 30% of the increase in score after the turn. This is possible only when there are less than two information tokens.

Assume a situation. If an agent has a card X that is known to be red, the agent can see the set of cards that X can be by looking at the Hanabi field, the set of discarded cards, and another player’s hand. For example, the other player has one red 2, there is one red 3 in the stack of discarded cards, and the red Hanabi of the field is 3. At this time, the set of cards which X can be is (Red 1, Red 1, Red 2 Red 4, Red 4, Red 5). After this turn, the probability of not needing X is 50%, because the cards involved in the score increase are Red 4 and Red 5. Therefore, “DiscardRiskyCard” can be used for X. The agent performs the same calculation for all cards, and the agent examines whether to discard the card with the highest probability of unnecessary.

#### PlayRiskyCard

The action of this function determines that an agent can play a card if the probability of success in playing the card in the hand multiplied by F is 30% or more. F is a parameter which varies depending on the number of failures of play at that time. The parameters are 100, 75, and 50 for 0, 1, and 2 failures.

Assume a situation. If an agent has a card Y whose number is 2 but whose color is unknown, the agent can see the set of cards that Y can be by looking at the Hanabi field, the set of discarded cards, and another player’s hand. For example, the other player has one Blue 2 and Red 2, there are one White 2 and one Yellow 2 in the stack of discarded cards, and the Hanabi of the field is (White 2, Red 1, Blue 3, Yellow 2, Green 0). At this time, the set of cards which Y can be is (Red 2, Green 2, Green 2). That is, the play success probability of card Y is 33%. Therefore, when the number of play failures is 0, “PlayRiskyCard” can be used for card Y, but when the number of play failures is 1 or 2, it cannot be used.

### Evaluation of Risk Sensitivity

In this section, we compare the responses of human participants to the agent with high risk sensitivity and responses to the agent with low risk sensitivity; these agents were implemented in the previous chapter. The value of the impression of the risk-taking agent was obtained from the difference in the participant’s impression of each agent. We then correlated each participant’s risk sensitivity and level of optimism with their impression of the risk-taking agent. From the correlation, we consider which characteristeics of participants make them likely to prefer risk-taking agents.

#### Hypothesis

This experiment was meant to determine whether the participants were satisfied with an agent with a risk sensitivity close to their own. We considered how agents taking risks affected the user’s impression. The hypothesis is as follows: the evaluation of an agent with a tendency similar to the player has a positive correlation with the degree of similarity.

#### Experimental Procedure

We first explained the rules of Hanabi and how to use the interface to play games. After that, the participants performed about 10 test plays to get used to the game. Then, participants played Hanabi twice with the above two kinds of agents and evaluated each agent. The order of agents played by the participants was counterbalanced. Participants played Hanabi using the interface on computer. Participants also answered a questionnaire about their own tendencies. At the end of the experiment, participants were interviewed on three points: “Did you recognize the differences between the two agents? And what was it like?”, “How was the usability of the Hanabi interface?” and “If you have any opinions, please tell me how you feel about the experiment.”

The participants were 30 undergraduate and graduate students aged 18–23 years. The study population consisted of 18 men and 12 women.

#### Evaluation Questionnaire

Participants answered questionnaires about the abilities of agents with which they played Hanabi. Participants also answered questions regarding their risk sensitivities in two specific questionnaires. The questionnaire regarding agents’ abilities is described as follows. All questionnaires were answered on a seven-point Likert scale.

• Questionnaire about agents’ abilities
  QA-1) I think this AI understands the rule of Hanabi
  QA-2) I understood the intention of this AI’s action
  QA-3) I felt consistency in the behavior of this AI
  QA-4) I think this AI works well with me
  QA-5) The AI’s behavior in each situation was predictable
  QA-6) I thought it was easy to play a game with this agent
  QA-7) I am satisfied with the result of this game with this AI

We also recommended that participants write comments about agents and gameplay.

In order to quantitatively assess participants’ risk-taking tendencies, two types of questionnaires were prepared and used: the General Risk Propensity Scale (GRiPS) developed by Zhang et al. (2019), and the Optimistic Scale by Scheier and Carver (1985). The optimistic scale measures an individual’s personality by focusing on two axes: optimism and pessimism. This evaluates whether the player is tolerant of his or her own mistakes and is widely used for the evaluation of participants in various experiments. GRiPS is a new evaluation measure for tasks, and is applied not only to specific situations, but also to general individual risk-taking tendencies, such as whether players take risks as a trade-off to achieve higher scores. This scale compensatively measures each participant’s internal state.

Each of these questionnaires, when the overall answers were examined, revealed a relative risk-taking tendency and relative optimism among subjects in the study group.

• Items of GRiPS:
  ➢ QG-1) Taking risks makes life more fun
  ➢ QG-2) My friends would say that I’m a risk taker
  ➢ QG-3) I enjoy taking risks in most aspects of my life
  ➢ QG-4) I would take a risk even if it meant I might get hurt
  ➢ QG-5) Taking risks is an important part of my life
  ➢ QG-6) I commonly make risky decisions
  ➢ QG-7) I am a believer of taking chances
  ➢ QG-8) I am attracted, rather than scared, by risk
• Items of Optimism Scale:
  ➢ QO-1) When it is not clear what the outcome will be, always consider the good side (optimistic item).
  ➢ QO-2) I can relax as I wish (filler item)
  ➢ QO-3) When I think that something is going wrong for me, it usually happens (pessimistic item).
  ➢ QO-4) I always think of the bright side of things (optimistic item)
  ➢ QO-5) I am very optimistic about my future (optimistic item)
  ➢ QO-6) I am blessed with many friends (filler item)
  ➢ QO-7) Keeping busy is important to me (filler item)
  ➢ QO-8) I don’t expect things to go well (pessimistic item)
  ➢ QO-9) Things never get the way I want them to (pessimistic item)
  ➢ QO-10) I am not upset easily (filler item)
  ➢ QO-11) I believe that “There is joy in the shadow of sorrow.” (optimistic item)
  ➢ QO-12) Things rarely go well beyond one’s wildest expectations (pessimistic item)

### Result

The hypothesis to be tested in this study was “people are likely to be more favorably impressed by agents which are relatively similar in risk-taking tendency than by a dissimilar agent.” The scores are 15.48 (SD 3.22) in high-risk condition and 15.43 (SD 4.42) in low-risk condition We applied Wilcoxon’s rank-sum test and there is no significant difference.

First, we determine whether there is a difference in the evaluation of the two kinds of agents, regardless of the character of the participants. In this study, the participants’ evaluation of the agents was considered as a relative evaluation between the high-risk and low-risk testers. Table 3 shows the result of examining whether there is a difference in each evaluation item between agents with high risk-taking tendencies (H) and agents with low risk-taking tendencies (L) for the experiment’s entire set of participants using the Welch *t*-test.

As shown in Table 3, significant differences were found for items QA-3. This suggests that the participants felt, on average, that the agents who did not take risks were more consistent. A possible reason for this is that the high risk-taking agents sometimes decided their actions probabilistically, but the threshold of the probability was not clear to the experimental participants, so it can be inferred that high risk-taking agents gave the impression that their actions were inconsistent. On the other hand, there was no significant difference in the evaluation items except for QA-3, so it can be concluded that there was little difference in impressions between the two agents. Therefore, it is necessary to confirm how participants’ evaluations of agents with different risk-taking tendencies differ according to each participant’s risk sensitivity.

Next, the basic data of the questionnaire on the characteristics of the participants are shown. For GRiPS, the average and standard deviation of the total are shown in Table 6 because the total represents participants’ risk-taking tendencies. For the optimism scale, the total of the optimistic items represents the respondent’s optimism, and the total of the pessimistic items represents the respondent’s pessimism. For the sake of simplicity, modified optimism is defined as the sum of the answers for the optimistic items minus the sum of the answers for the pessimistic items; the higher the modified optimism, the more optimistic the participant. The means and standard deviations are shown in Table 6.

**TABLE 6:** Mean and SD of evaluations of participants.

	Mean	SD
GRiPS	3.28	0.83
Optimism scale	−0.42	4.99

We also applied Pearson’s correlation analysis for the data. Table 7 shows that there was a moderate correlation for some items. In Table 5, with the exception of question item QA-3, not significant difference was found in the evaluation of the two kinds of agents among all participants, while the results of Table 7 reveal that the evaluation of the agents of participants differed depending on individual differences.

**TABLE 7:** Correlation coefficients (N: 30)^*^ >0.40, ^+^<−0.40.

Q on agents	Correlation coefficient (relative evaluation-GRiPS) with *p*-value		Correlation coefficient (relative evaluation-optimism scale) with *p*-value	
QA-1)	−0.28	0.13	−0.01	0.94
QA-2)	0.00	0.98	0.14	0.45
QA-3	−21	0.25	−0.14	0.45
QA-4	0.12	0.52	0.05	0.78
QA-5	0.30	0.10	0.37	0.04*
QA-6	0.11	0.57	0.09	0.64
QA-7	0.09	0.63	−0.06	0.77

Table 7 shows the results of measuring the two correlation coefficients between the relative evaluation values of the two kinds of agents and the participants’ risk-taking characteristics. One of these was measured by GRiPS; the higher the correlation coefficient, the more a risk-taking participant appreciated the high risk-taking agent in the questionnaire items. Another was measured by modified optimism; the higher the correlation coefficient, the more an optimistic participant appreciated the high risk-taking agent in the questionnaire items.

## Discussion

### Self-Estimation

The results of the experiment indicated significant differences and trends with regard to the S′ and D conditions, and the D and H conditions for Q3–Q6 for the impression evaluation item. There were no significant differences between the S′ and H conditions. GQS, Q3, and Q4 correspond to the likability of the agent, and Q5 concerns the intelligence of the agent. Accordingly, the agent accurately performs blind self-estimation so that the player can perceive it as a human being.

It was discovered that a sense of intimacy and intelligence was felt with the agent in the I condition to the same extent as with a human teammate. In addition, when performing blind self-estimation accurately, it can be said that the agent was able to respond to the request of the player to the same extent as a human teammate.

To investigate the relationship between the impression evaluation items and the success rate of playing the estimated card, a Pearson’s correlation analysis was performed on the S condition. The results showed a positive correlation between Q1 and Q7 and the play success rate (Table 8). In the self-estimation strategy, the agent accurately estimates its self-status using the teammate’s signals. Furthermore, in this case, the player perceived humanity, affinity, intelligence, and good communication in the agent.

**TABLE 8:** Correlation between play success rate and impression evaluation items of cards from estimated information**: *p* <0.001^*^: *p* <0.05.

	Pearson correlation coefficient	Significance probability
Q1	0.514	0.010*
Q2	0.462	0.023*
Q3	0.651	0.001**
Q4	0.489	0.015*
Q5	0.672	0.000**
Q6	0.615	0.001**
Q7	0.577	0.003**
Q8	0.223	0.296
Q9	0.270	0.201
Q10	0.023	0.914
Q11	0.072	0.739
Q12	0.404	0.050

#### Score and Thinking Time

The results of the evaluation experiment showed that the significance of the score regarding the deterministic strategy of self-estimation could not be confirmed. Therefore, to investigate the relationship between the score and other evaluation items, a correlation analysis was performed on the S condition. The findings indicated a positive correlation between the play success rate of the card and the score (*r* = 0.662, *p* < 0.01). Using this information, we also found that the score was significantly increased by the agent’s accurate blind self-estimation in the self-estimation strategy.

There was no significant difference in the scores between the subjects’ average thinking time and the deterministic self-estimation strategy. Therefore, to investigate the relationship between the average thinking time and other evaluation items, correlation analysis was performed for the S and D conditions. The results of this analysis showed that when the agent used the self-estimation strategy, the average thinking time and impression evaluation item Q11 (i.e., whether the game was enjoyed on an individual basis) were positively correlated (*r* = 0.533, *p* < 0.01). In addition, when the agent used the deterministic strategy, a negative correlation was noted between the average thinking time and the impression evaluation item Q10 (i.e., regarding failed behavior) (*r* = −0.412, *p* < 0.05). Thus, it can be said that subjects were more likely to feel confident about their actions and believe that they contributed to the game when more time was available to consider possible actions.

#### Failure of Self-Estimation

A game between agents was simulated 100 times each for all the self-estimation strategy and the deterministic strategy. The card estimation average was 5.2 (SD: 2.8) per game and the average estimated ratio was 84% (SD: 21) per game for the self-estimation strategy. The results of the *t*-test regarding the estimated number of turns and estimated success rate of the card game between this simulation and the S condition showed no significant difference for the estimated number of turns (*p* > 0.1), but the estimation success rate was significantly lower (*p* < 0.01).

The reason for the estimated success rate being significantly lower in the simulated S condition for the simulation can be attributed to the fact that even if the impression evaluation item (Q13 or failure to play the correct cards when given information) was presented, the teammate played card I, focusing on his/her own opinion. This aspect was uncovered during the interviews. When humans performed actions not found in the teammate’s behavior simulation when using the self-estimation strategy, it was confirmed from the game record that the card estimation was erroneous. For example, if only one type of card in a deck (e.g., all the cards have the number 5, and cards with numbers 1 and 2 cards have already been discarded) are in the teammate’s hand, information on that card is presented. In this case, the self-presumptive strategy presumed that the presented card was a playable card, and thus, it was played. When a difference exists between the behavior of the subject and the simulation model of their partner’s behavior in the self-estimation strategy (i.e., the behavior principle of the agent differs from that of the human), the accuracy of the blind self-estimation decreases. We believe that this problem can be solved by creating a behavior model that resembles a human’s choices of actions.

### Thinking Time

Because there was no difference in the scores for the P condition compared with the C and R conditions, the agent developed in this study that changes the thinking time according to the priority of the action does not affect the scores when compared with either the constant thinking time agent or the random thinking time agent. There was no difference in the scores, however, there was a difference in the impression of the agents.

#### Discussion for Comparison With Constant Thinking Time

Since there was no significant difference in the scores between the two agents, the agents developed in this study that change the thinking time do not affect the scores in comparison with agents with constant thinking time.

According to Table 1, agents who change their thinking time according to their actions give players the impression that they are worried. It is considered that the reason for this is that the agent in the P condition is distressed by a long time of suffering when the agent takes a low-priority action. However, only 8 out of 20 players noticed the difference in their thinking time, so some players may have felt that they were unconsciously worrying.

Of the 12 players who were unaware of the difference in thought time, 8 responded to a questionnaire on algorithm differences, such as “Most of the time, they gave me easy-to-understand information that led me to play.” “I felt there was a lot of disposal activity.”, despite the fact that there was no difference in algorithm between the 2 agents. This is thought to be because some situations in which the behavior of an agent is biased due to the hand or the board in the game are judged to be the tendency of the behavior of the agent.

Table 4 also shows that players who notice the difference in their thinking time are smart and friendly about agents who change their thinking time. This is thought to be because, in the behavior that is apt to be troubled in the game between humans, the player notices that the agent is troubled and gives the player the impression that he is willing to think carefully and cooperate with the same viewpoint as the human. Table 4 also shows that players who notice the difference in thinking time feel comfortable playing games with agents that change their thinking time. This may be because they felt human towards the agent and could play the game without feeling tense.

#### Discussion for Comparison With Constant Random Time

Since there was no significant difference in the scores between the two agents, the agent developed in this study that changes the thinking time does not affect the scores in comparison with the random thinking time agent.

According to Table 5, we found that agents that change their thinking time according to their action priorities are smarter than agents that change their thinking time randomly, and that they have a better understanding of their action intentions. This may be because longer thinking time in low-priority activities, such as random abandonment or information provision that does not lead to play, gives players the impression that they are more likely to think and choose actions than agents in random thinking time.

It was suggested that the agent who changes the thought time randomly felt that it was more troubled than the agent who changes the thought time according to the priority of the action. This may be because, in a random thinking time agent, when a player knows that “Agents can play specific cards”, that is, when a behavior selection is determined in a short time, the agent’s thinking time is longer than usual, and the player feels that there is more time to worry than usual. In the case of a random thinking time agent, it is possible that the thinking time is short when the playable card is not known, that is, when it is difficult to decide the action in a short period of time, but in such a case, it is considered that the thinking time is not short because the agent feels that the action can be decided in a short period of time by information that is not visible from the player side, such as the player’s hand cards.

#### Summary for Thinking Time

Regarding the item “Q1. Did you feel that the opponent was worried a lot?”, the P condition was significantly higher than the C condition, and the R condition was significantly higher than the P condition. From this, it can be seen that the agent developed in this study, which changes the thinking time according to the priority of the action, gives the player an impression that the agent is distressed compared to the agent with constant thinking time, or not distressed compared to the agent with random thinking time.

Regarding the item “Q3. Did you feel the opponent friendly?” “Q6. Do you feel familiar with the game?”, the P condition was significantly higher than the C condition, but there was no difference between the R and P conditions. From this, it is considered that only the factor of the change of thinking time every time affects the familiarity of the agent and the familiarity to the game, and that the change of thinking time with meaning according to the action does not affect the familiarity and the habituation.

With regard to the item “Q2. Did you think that the opponent is clever?”, among the players who noticed the difference in thinking time, the P condition was significantly higher than the C and R conditions. This indicates that the agent developed in this study, which changes the thinking time according to the priority of the action, gives the player the impression that it is smarter than the agent with constant thinking time or random thinking time. In other words, the agent developed this time can show the tacit cooperative attitude to the other party from the fluctuation of the length of the thinking time compared with the conventional agent.

As described above, in designing agents to cooperate with humans, it is possible to convey an implicit cooperative attitude and make them feel intelligent by changing the length of the agent’s thinking time according to their actions, and by implementing this function, it is thought to lead to the development of agents that are easier to cooperate with humans.

### Risk Tendency

#### Correlation Between the Relative Evaluation of Agents and GRiPS

The similarity of player’s risk sensitivity and agent’s risk sensitivity measured by GRiPS did not support our hypothesis. The improvement of player’s satisfaction with the game and favorable impression of the agent was not clarified. Next, in order to deepen the discussion on Hypothesis 1, we consider the correlation between Modified Optimism, which is the inner state of tolerance or optimism to mistakes of players in games, and evaluation for agents taking risks.

#### Correlation Between Relative Evaluation of Agents and Modified Optimism

The correlation coefficient between the relative evaluation of agents and the Modified Optimism show a moderate correlation in the question item QA-5. The more optimistic the participants, the more likely they were to believe that the risk-taking behavior of AI was more predictable.

This result particularly supports hypothesis 1. This result means that the players have different game results depending on the risk sensitivity of game agents. Players are also more likely to think that agents with similar risk tendencies are more willing to cooperate. This has important implications for the implementation of game agents. The designer of the game agent could make user’s better impression with our method.

## Contribution

Our contribution is that we determined that players have a better impression of agents with self-estimation, and thinking time similar to those of the players when playing Hanabi. We speculate that these findings can be applied to games that have the same features as Hanabi regarding self-estimation and thinking time. We also found several significant differences that are related with risk-sensitivity. When a user plays with an agent having a high risk, the user determines that the action of the agent is consistent. The more optimistic the player, the more predictable the behavior of the high-risk agent. Behavioral consistency and predictability are behaviors that trigger users to think they understand the agent. We believe that an agent’s risk-taking behavior enhances the agent’s personality, especially for optimistic players. We think that it is possible to implement agents in a game with such features using the knowledge of this research. Several cooperative games have coordination problems for players. There is a tradeoff in risk-taking behavior. For example, in the game “The Mind” by Wolfgang Warsch (Warsch, 2019), players need to play the cards with the smallest numbers in order in their hands without communication. Playing a higher-numbered card has a higher risk. If the risk-taking tendencies of the players do not match, the game is lost. The requirements for the agents in this game are estimated to be similar to those for Hanabi players. Thus, we believe that our approach is applicable to this task. These coordination problems are also a major problem not only in the field of games, but also in general multi-agent coordination. We also hope to determine how our method can be generalized to different tasks, not only games.

## Limitation

Our implementation and experiment still have four limitations: diversity of participants, evaluation factors for participants, limited targets, and long-term effects for participants.

Verification for a wide range of age groups: Although our results concern general knowledge of human behavior, all participants in this study were students in their twenties. Basically, human cognitive processing speed decreases with age. Therefore, it is assumed that there is a difference in impression depending on age. However, Hanabi’s rules are simple and easy to understand for any age group, so it’s hard to imagine any major differences between the conditions. Basically, human cognitive processing speed decreases with age. We will look at a wider age range to determine if there is any bias across age groups. In our experiment, we evaluated participants using offline human experiments because we can observe participants rigorously and track their behaviors in detail. In addition, due to resource limitations, our first experiments were performed only on male subjects. While the given task appears to be one that is likely to be unaffected by the player’s gender, experiments with female players are warranted in the future. Based on our study, we plan to conduct a future experiment through an online study using crowdsourcing.

Devising criteria to evaluate suitable user characteristics to match users and agents: In this study, we used GRiPS and the Optimism Scale to assess participants’ characteristics; these scales are generic and more appropriate measures are required to better match players and agents. Alternatively, it could be useful to measure user tendencies as they interact. Also, we need to be carefully research that founded significant difference is not because of fluke in future study, because of similarity on questions QA-1 to 7. At least, GRiPS does not have significant difference on this study, so we need careful evaluation method for future study in risk sensitivity. If game designers want to improve the user experience by considering an agent’s risk-taking tendency, they need to make players aware of the agent’s risk-taking tendency. In Hanabi, the difference in the risk-taking tendency could be expressed in terms of the probability that could be calculated from the state of the field. However, it is not always possible to express the same in other games.

Examination of contributions to long-term motivation: Because this is a short-duration study, the results of this study do not provide knowledge about the effects of repeating interactions over long periods. The benefits of similarity between users and agents in the short term may not be the same as those in the long term.

## Conclusion

In this research, we determined humans’ impressions of agents by imitating the cognitive function of estimating incomplete information from simulations of other players’ observations/information. The results of the experiments, which used the cooperative game Hanabi, showed that the more accurate the information estimation of the agent’s self, the more likely the agent’s partner is to perceive humanity, affinity, intelligence, and communication skills in the agent. These aspects led to higher game scores. In particular, when the agent’s own information estimation succeeded with high probability, it was confirmed that the sense of intimacy, intelligence, and communication skills that the other party feels toward the agent is as high as that in the case of a human teammate. However, in cases where the agent made an incorrect estimation because of a difference between the behavioral model of the teammate and the behavior of the human, we observed that human beings do not perceive humanity in agents.

In the thinking time experiment, the scores did not change as the agent’s thought time changed. However, it has been found that agents that change the length of their thinking time according to the priority of their actions give players the impression that they are distressed compared to agents with a certain amount of thinking time. In addition, by noticing the difference in thinking time, it was found that agents that change the length of thinking time according to the priority of actions give the impression that they are smarter than agents with a fixed thinking time or agents that randomly change thinking time. From this, it can be said that the agent that changes the thinking time according to the priority of the action shows the tacit cooperative attitude to the partner due to the change of the thinking time and is easier to cooperate than the conventional agent. Future prospects include more realistic changes in the length of thinking time. In this experiment, the normal thinking time was set at 3.5 s, and the thinking time was extended to 7.5 and 9.5 s for low-priority actions. However, since the actual human thinking time is affected not only by the priority of actions but also by various factors such as the complexity of the face and the comprehensibility of the partner’s strategy, implementing a strategy to change the thinking time by incorporating these factors is expected to lead to the development of agents that can more easily cooperate with humans.

We investigated the relationship between the concordance of the risk-taking tendencies of players and agents, the player’s impression of agents, and the game experience. When a user plays with an agent having a high risk, the user determines that the action of the agent is consistent. The more optimistic the player, the more predictable the behavior of the high-risk agent. Behavioral consistency and predictability are behaviors that trigger users to think they understand the agent. These results indicate that the evaluation of agents with different risk-taking tendencies varies among players. This study is also related to general risk sensitivity and tolerance to the partner agent during games. Therefore, this study suggests that game agent designers can improve the player’s disposition toward an agent and the game experience by adjusting the agent’s risk-taking tendency according to the player’s personality and thoughts during the game.

## Data Availability

The original contributions presented in the study are included in the article/Supplementary Material, further inquiries can be directed to the corresponding author.
